# A 71-year-old male with a life-threatening recurrence of hemolytic anemia, thrombocytopenia, and acute kidney injury after pembrolizumab therapy: a case report

**DOI:** 10.1186/s12877-023-04181-w

**Published:** 2023-08-09

**Authors:** Xin Zhang, Bi-xia Gao, Cui-yan Guo, Tao Su

**Affiliations:** 1 Renal Division, Department of Medicine, Peking University First Hospital, Beijing, 100034 People’s Republic of China; 2 Institute of Nephrology, Peking University, No.8 Xishuku Street, Beijing, Xicheng District, 100034 People’s Republic of China; 3 Key Laboratory of Renal Disease, Ministry of Health of China, Beijing, 100034 People’s Republic of China; 4 Key Laboratory of CKD Prevention and Treatment, Ministry of Education of China, Beijing, 100034 People’s Republic of China; 5 Research Units of Diagnosis and Treatment of Immune-mediated Kidney Diseases Chinese Academy of Medical Sciences, 100034 Beijing, PR China; 6 Department of Pulmonary and Critical Care Medicine, Peking University First Hospital, Beijing, 100034 China

**Keywords:** Pembrolizumab, Autoimmune hemolytic anemia, Thrombocytopenia, Diarrhea, Lung adenocarcinoma, Acute kidney injury

## Abstract

**Background:**

Immune checkpoint inhibitors (ICIs) have revolutionized cancer treatment. However, their use has been restricted in patients with preexisting autoimmune diseases due to concerns about increased risk of immune-related adverse events (irAEs).

**Case presentation:**

We present a case of a patient with stage IV lung adenocarcinoma and a history of complement-mediated autoimmune hemolytic anemia in remission. After receiving a single dose of pembrolizumab, the patient experienced life-threatening recurrent hemolytic anemia, de novo thrombocytopenia, diarrhea, myocarditis, and acute kidney injury. Laboratory tests confirmed the diagnosis of Evan's syndrome, with positive PAIgG and direct antiglobulin test. Treatment with intravenous methylprednisolone at a dose of 2 mg/kg resulted in a favorable response, with resolution of symptoms and rapid recovery of kidney function. The probable cause of pre-renal hypoperfusion (evidenced by a BUN-to-creatinine ratio of 48.1) leading to acute tubular injury was attributed to pembrolizumab-induced diarrhea.

**Conclusions:**

This case illustrates a life-threatening recurrence of complement-mediated autoimmune hemolytic anemia induced by ICIs. Clinicians should carefully consider the expected efficacy and potential toxicity before initiating ICIs therapy in patients with preexisting autoimmune diseases. Additionally, the occurrence of acute kidney injury during ICIs therapy adds complexity and requires careful differential diagnosis.

## Background

In recent years, immune checkpoint inhibitors (ICIs) have emerged as the standard treatment for various types of cancer, significantly improving overall patient survival rates. However, these inhibitors can also elicit immune responses against self-antigens, resulting in a range of immune-related adverse events (irAEs) [1]. To mitigate the risk of such adverse events, clinical trials involving ICIs have typically excluded patients with preexisting autoimmune diseases [2]. Nevertheless, the impact of preexisting autoantibodies on the safety and efficacy of ICIs in cancer patients remains poorly understood. This report details a case of a patient with stage IV lung adenocarcinoma and autoimmune hemolytic anemia (AIHA), who experienced hemolytic anemia, thrombocytopenia, diarrhea, myocarditis, and acute kidney injury following treatment with pembrolizumab.

## Case presentation

**Fig. 1:**
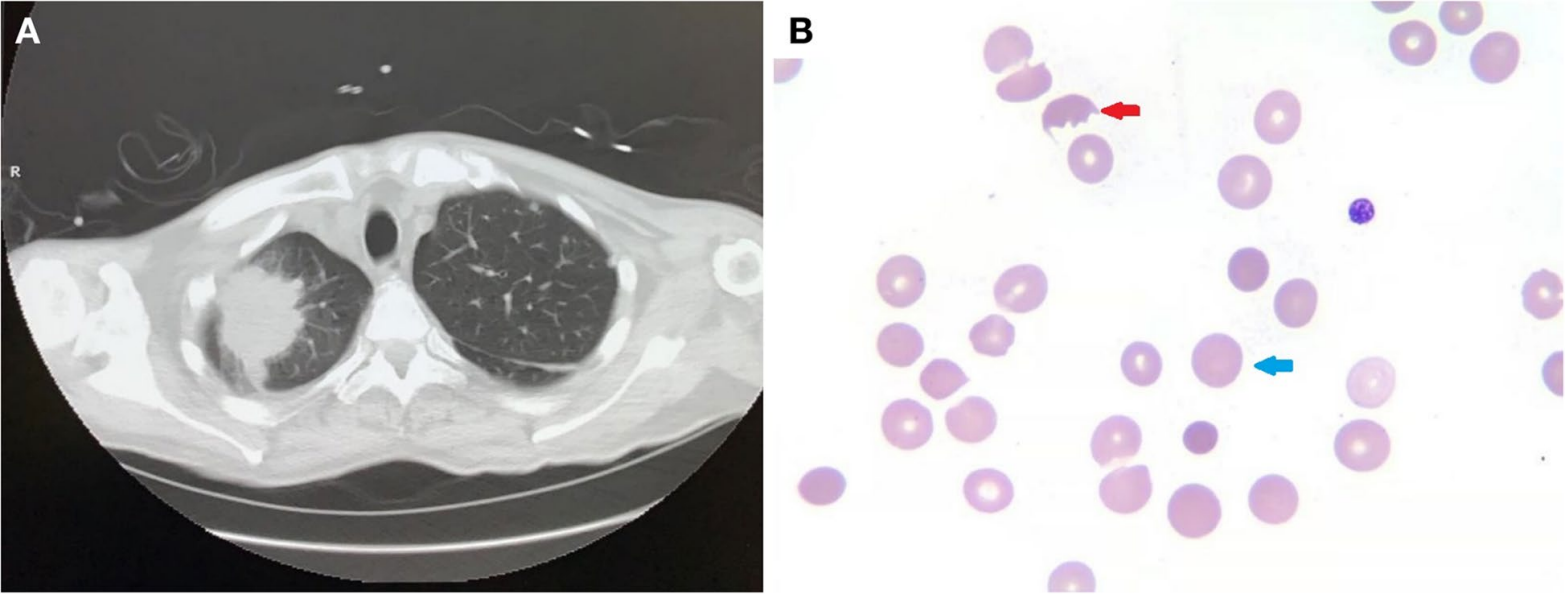
**A** the CT scan showed the tumor at the right upper lobe of the right lung, **B** The schistocytes (red arrow) and spherocytes (blue arrow) could be seen under this field of vision in peripheral blood smear

A 71-year-old male was diagnosed with stage IV lung adenocarcinoma (Fig. 1) and concurrent complement-mediated AIHA five months prior to referral. The TNM stage at the time of diagnosis was classified as T4N3M1a, indicating a locally advanced tumor with distant metastases. The tumor grade of the lung cancer was also determined to be Stage IV, indicating widespread disease. Needle biopsies of the right lung and right axillary nodes confirmed the presence of adenocarcinoma, but further information regarding the specific histopathological subtype was not obtained. Subsequent PET-CT imaging revealed involvement of multiple organs, including both lungs, lymph nodes in the right axilla, right hilum and mediastinum, supraclavicular fossa, and a possible involvement of the right kidney. The patient's ECOG performance status was determined to be stage 2, suggesting a moderate restriction in daily activities with the ability to care for oneself. However, the evaluation of the patient's nutritional status was incomplete with the absence of NRS-2002 or PG-SGA assessments in the medical records, despite a BMI value of 19.8. The patient demonstrated responsiveness to intravenous immunoglobulin and daily prednisone at a dose of 1mg/kg. As a result, his hemoglobin level stabilized within the range of 110-120g/L, and a normalized direct antiglobulin test (DAT) was observed. Over the following two months, the prednisone dosage was gradually tapered off. After consultation with a hematologist and obtaining fully informed consent about the risk of AIHA recurrence, the patient opted for immune therapy instead of chemotherapy. However, ten days after receiving a single dose of pembrolizumab (200mg), the patient experienced an acute onset of symptoms including diarrhea, nausea, and vomiting. Due to the presence of fever, fatigue, hypersomnia, and oliguria, the patient was referred to the emergency room .

Upon admission, the patient appeared conscious but weak and displayed a pale appearance. Vital signs were as follows: T 38.7℃, BP 101/60mmHg, HR 133 bpm, RR 30 bpm, and oxygen saturation at 97%. The bilateral lungs were coarse, with no rales on auscultation. His heart rate was regular, with no pathological murmur in any valve auscultation area, and the abdomen was soft with no tenderness. He had no skin rashes and peripheral edema. Laboratory test results, summarized in Table 1, suggested severe hemolytic anemia upon admission, with a hemoglobin level of 34 g/L (normal range: 120-150 g/L), a reticulocyte count of 4.42% (normal range: 1.0-2.5%), a total bilirubin level of 56.1 μmol/L (normal range: 1.7-20 μmol/L), an unconjugated bilirubin level of 41.2 μmol/L (normal range: 1.7-14 μmol/L), and an elevated LDH level of 647 IU/mL (normal range: 100-240 IU/mL). Thrombocytopenia was observed from day 2 onwards, with a platelet count of 72 x 10^9^/L (normal range: 125-350 x 10^9^/L). The patient displayed significantly increased liver enzyme levels, with ALT at 460 IU/mL (normal range: 7-40 IU/mL) and AST at 579 IU/mL (normal range: 15-35 IU/mL), as well as elevated myocardial enzyme levels including CK-MB at 19.4 ng/mL (normal range: <5 ng/mL) and cTnI at 16920.3 ng/L (normal range: 0-11.6 ng/L). Serum creatinine was measured at 292.8 μmol/L (normal range: 44-133 μmol/L) and BUN at 31.01 mmol/L (normal range: 1.8-7.1 mmol/L). Cold agglutinin testing yielded negative results. The complement C3 level was 0.358 mg/L (normal range: 0.6-1.5 mg/L). The direct antiglobulin test (DAT) revealed the presence of 2+ positive anti-IgG and anti-C3, confirming a diagnosis of recurrent AIHA. Furthermore, the platelet-associated IgG was found to be positive at a level of 10% (normal range: <10%). Ultrasonography detected newly emerged deep vein thrombosis in the lower extremities, indicating a hypercoagulable state. The d-dimer level was measured at 11.13 mg/L (normal range: <0.24 mg/L [DDU]), while the prothrombin time (PT) was relatively normal at 13.2 seconds (normal range: 10.1-12.6 seconds), the activated partial thromboplastin time (aPTT) was 32.2 seconds (normal range: 26.9-37.6 seconds), and the fibrinogen level was 3.2 g/L (normal range: 2-4 g/L).

**Table 1:** The laboratory findings of the patient

Items	Day 1	Day 2	Day 7	Day 16
WBC (× 10^9^/L) (3.5–9.5)	25.02	22.14	8.8	6.9
Hb (g/L) (120–150)	34	48	78	76
PLT (× 10^9^/L)(125–350)	420	72	57	219
Ret (%) (1.0–2.5)	4.42			1.5
ALT (IU/L) (7–40)	13	460	19	25
AST (IU/L) (15–35)	28	579	21	17
LDH (IU/L) (100–240)	647	1846	1608	443
cTnI (pg/ml) (0–11.6)	23.5	16,920	874.3	13.5
CK-MB (ng/ml) (< 5)	1.5	19.4	3.55	2.8
Scr (μmol/L) (44–133)	106	292.8	418.4	149.1
BUN (mmol/L) (1.8–7.1)	20.4	31	44.65	18.86
D-Dimer (mg/dl) (< 0.24)	0.97	11.13	7.83	2.66
CRP (mg/L) (0–3)	63	86	37	6

The patient was diagnosed with Evan's syndrome, characterized by AIHA with immune-related thrombocytopenia as an adverse effect induced by immunotherapy. The differential diagnosis considered complement-mediated thrombotic microangiopathy, antiphospholipid syndrome, and disseminated intravascular coagulation (DIC). However, a peripheral blood smear showed infrequent presence of schistocytes (0.7%) and spherocytes (0.4%) (Fig. 1B). Additionally, the levels of CH50, complement factor H, and ADAMTs13 activity were within normal range (72% [44-99%]). Further examination revealed that autoantibodies including anti-complement factor H, antinuclear, rheumatoid factor, antiphospholipid, anti-glomerular basement, and anti-neutrophil cytoplasmic antibodies were all within normal ranges. Despite the significantly elevated d-dimer, the relatively normal prothrombin time (PT), activated partial thromboplastin time (aPTT), and fibrinogen levels did not support the diagnosis of DIC.

**Fig. 2:**
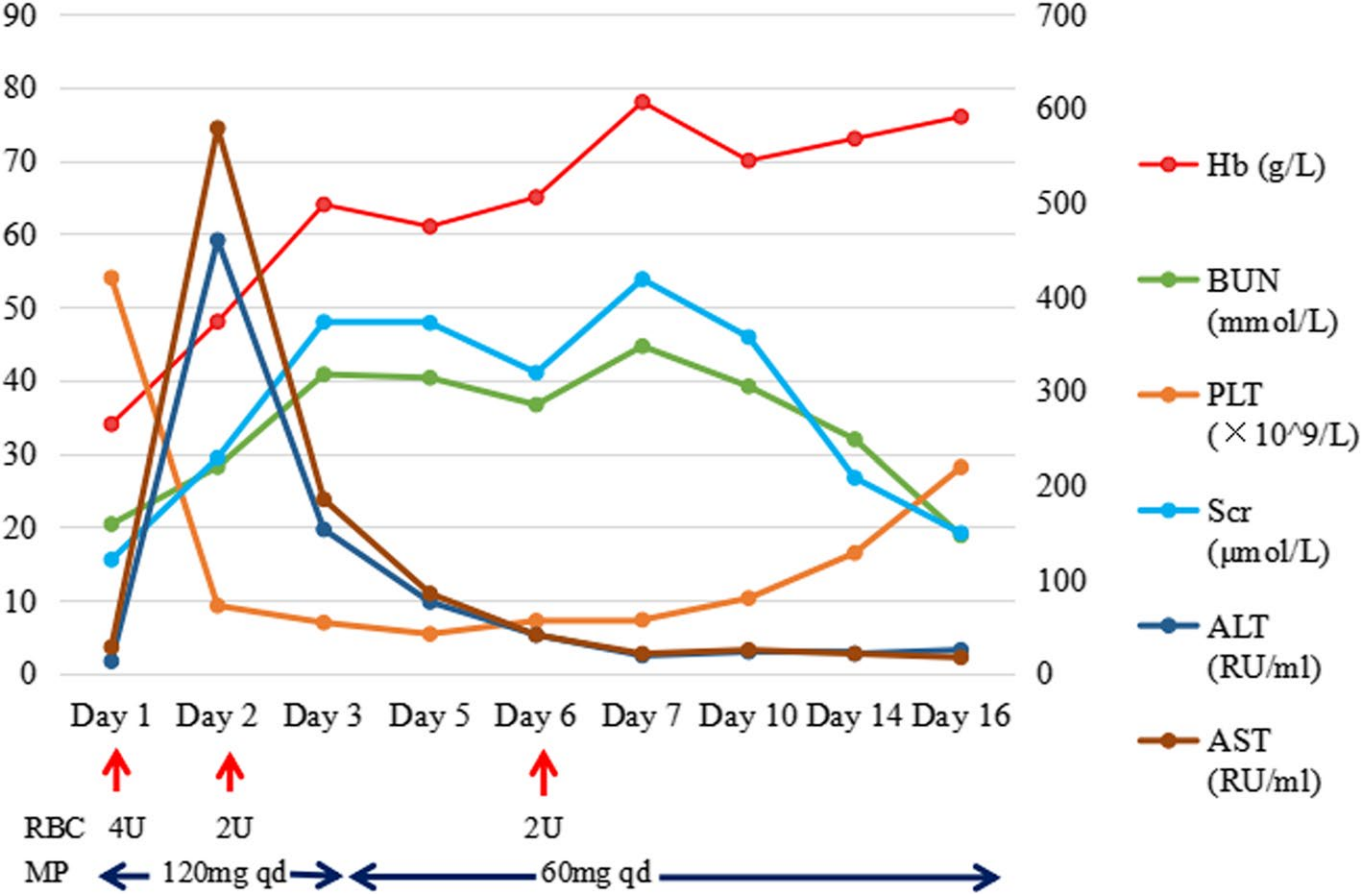
The clinical course of the patient

The patient's renal function deteriorated despite receiving volumetric resuscitation. A routine urinalysis showed no signs of proteinuria, leukocyturia, or hematuria. His diarrhea improved spontaneously. To treat the AIHA, the patient was administered daily intravenous methylprednisolone at a dose of 120mg (2mg/kg) for three days, followed by oral prednisone at a dose of 60mg. Within 16 days, the patient's hemolysis, thrombocytopenia, abnormal cardiac and liver enzymes, as well as kidney function, rapidly recovered as shown in Table 1 and Fig. 2. However, the patient declined a kidney biopsy. Upon discharge from the hospital, the patient's serum creatinine level was 149.1μmol/L. After consulting with the respiratory doctor, the patient and their family opted for palliative care and decided not to pursue further treatment for the lung cancer. Unfortunately, the patient passed away within three months of being discharged from our department. In conclusion, the patient presented with pembrolizumab-induced Evans syndrome, diarrhea, myocarditis, and acute kidney injury (AKI). Considering the rapid restoration of renal function within a few days, the most likely pathological diagnosis was acute tubular necrosis.

## Discussion and conclusions

This patient, diagnosed with stage IV lung adenocarcinoma, experienced multiple immune-related adverse events following pembrolizumab therapy. The irAEs observed included AIHA, thrombocytopenia, acute kidney injury (AKI), myocardial injury, and hepatic abnormalities, which require further analysis. Based on the patient's history of complement mediated AIHA, the sudden occurrence of recurrent hemolysis with hypocomplementemia was suspected to be induced by pembrolizumab, which targets the programmed cell death 1 (PD1) receptor and activates T lymphocytes. The presence of positive platelet-associated IgG (PAIgG) indicated Evans syndrome, a concurrent autoimmune condition characterized by both positive DAT and positive PAIgG. Another potential diagnosis considered was thrombotic microangiopathy (TMA), given the presence of hypocomplementemia, hemolytic anemia, and oliguric AKI, previously associated with PD-1 inhibitors. However, the possibility of TMA was ruled out due to asynchronous occurrence of hemolytic anemia and thrombocytopenia, as well as the absence of schistocytes in the peripheral blood smear (less than 2%). Based on the analysis conducted, the worsening renal function was more likely attributed to pre-renal hypoperfusion (as evidenced by a BUN-to-creatinine ratio of 48.1) caused by pembrolizumab-induced diarrhea. Additionally, although complement-mediated hemolytic anemia and thrombocytopenia are commonly associated with catastrophic antiphospholipid syndrome (CAPS), the patient tested negative for antiphospholipid antibodies, ruling out CAPS as a diagnosis. Disseminated intravascular coagulation (DIC) was also excluded as a diagnosis, as the patient's prothrombin time (PT), activated partial thromboplastin time (aPTT), and fibrinogen levels were relatively normal.

Autoimmune hemolytic anemia (AIHA) is a rare paraneoplastic syndrome associated with solid tumors, characterized by increased destruction of red blood cells (RBCs) due to the presence of anti-erythrocyte autoantibodies, with or without complement activation [3]. Secondary AIHA accounts for approximately half of the cases and is more commonly seen in B-cell malignancies, autoimmune diseases, and drug-related causes. It has also been reported in association with renal cell cancer, ovarian cancer, thymus cell cancers, and Kaposi sarcoma [4]. The onset of AIHA can precede the diagnosis of cancer, occur simultaneously, or present after cancer treatment. In this particular patient, we hypothesized that the AIHA was primarily mediated by autoimmune mechanisms triggered by lung adenocarcinoma. A positive DAT result is the definitive clinical indicator of AIHA. The pathological and clinical features of AIHA are influenced by the autoantibody class, thermal amplitude, and their ability to activate complement. It is believed that the presence of sufficient C3d deposits on the erythrocyte membrane leads to visible agglutination through cross-linking of RBCs [5]. Some patients may have additional antibodies, such as platelet antibodies, resulting in the development of Evans syndrome, as observed in the present patient.

Previous studies have suggested that AIHA is associated with an increased risk of thromboembolic events [6]. In line with the literature, we attributed the occurrence of newly-onset deep vein thrombosis (DVT) in our patient to AIHA. The prothrombotic state observed in AIHA stems from an imbalance between complement activation and regulation on host surfaces, leading to resistance to anticoagulant and/or antiplatelet therapies. In our patient, the presence of significantly elevated levels of D-dimer, myocardial enzymes (CK-MB and cTNI), and C-reactive protein indicated a systemic inflammatory condition. Therefore, in addition to the combination of steroids, rituximab, and/or intravenous immunoglobulin (IVIG) [7], targeted therapies such as eculizumab, which inhibits complement system-mediated damage to RBCs, hold promise for the treatment of refractory AIHA [8, 9]. However, in the early stages of cancer, curative resection may provide an effective approach to achieve and sustain remission in AIHA.

Pembrolizumab and other immune checkpoint inhibitors can cause hematological irAEs such as AIHA. The incidence of anemia, thrombocytopenia, and neutropenia in patients treated with ICIs has been reported as be 9.8%, 2.84%, and 1%, respectively [10]. As with other immune-related adverse events, the standard approach to managing ICI-induced AIHA involves discontinuing immunotherapy and initiating glucocorticoid therapy. If glucocorticoid therapy is ineffective, additional immunosuppressive agents may be used. Although most patients with AIHA caused by immune-related adverse events have responded to glucocorticoid therapy according to previous reports, there are still cases of treatment failure [11–13]. Evans syndrome is a rare condition that poses greater treatment challenges compared to isolated AIHA. It is less responsive to standard therapies and associated with more frequent relapses and higher mortality rates [14, 15]. In addition to AIHA, the patient in this case also presented with other manifestations of irAEs, including diarrhea, myocarditis, and AKI. While AKI related to immune checkpoint inhibitors (ICIs) is not extensively discussed in the literature, its incidence is likely underestimated and estimated to be around 2.2 to 5% [16]. Several factors increase the risk of ICI-associated AKI, such as lower baseline estimated glomerular filtration rate, proton pump inhibitor use, and the presence of extra-kidney irAEs [17]. Acute interstitial nephritis, primarily driven by T cell-mediated immunity, is the most common clinical and pathological presentation. However, literature reports have also noted biopsy-proven acute tubular necrosis, immune complex glomerulonephritis, and thrombotic microangiopathy [16]. A kidney biopsy is crucial for accurately diagnosing patients with suspected renal irAEs, particularly those presenting with high-grade proteinuria, hematuria, presence of white blood cells/red blood cells/granular casts, or renal tubular acidosis (RTA). In the case of the current patient, a kidney biopsy was recommended, but the patient and their family declined due to rapid improvement in kidney function and the patient's estimated lifespan of a few months. Based on the analysis, the deterioration of renal function in this patient can be attributed to pre-renal hypoperfusion resulting from pembrolizumab-induced diarrhea. The most probable pathological diagnosis is acute tubular injury.

Patients with preexisting autoimmune diseases and malignant tumors have been excluded from clinical trials involving immune checkpoint inhibitors due to concerns about potential exacerbation of autoimmune diseases. However, retrospective data from patients with autoimmune diseases and concomitant malignancies treated with ICIs suggest that although there is an increased risk of irAEs, the baseline autoimmune disease does not significantly worsen clinical outcomes in cancer patients receiving ICIs [18–21]. Nonetheless, it is important to consider the potential for severe flares of preexisting autoimmune diseases before initiating ICIs, considering the expected efficacy of treatment. Case series in the literature showed that only a small number of patients experienced a recurrence of AIHA when subjected to immunotherapy rechallenging. This suggests that the reintroduction of therapy could be cautiously considered if deemed suitable. However, ongoing prospective clinical studies focusing on ICIs in autoimmune patients may provide further insights into the complex relationship between irAEs and preexisting autoimmunity.

In conclusion, this case represents a typical instance of life-threatening recurrent complement mediated AIHA induced by ICIs. A thorough assessment of the potential toxicity of ICIs is necessary for patients with advanced cancers and preexisting autoimmune diseases. The development of acute kidney injury during ICI therapy in this case complicated the situation and necessitated a cautious approach to differential diagnosis.

## Data Availability

The original contributions presented in the study are included in the article. Further inquiries can be directed to the corresponding authors.

## Abbreviations

ICIs: Immune checkpoint inhibitors
AIHA: Autoimmune hemolytic anemia
irAEs: Immune-related adverse events
AKI: Acute kidney injury
DAT: Direct antiglobulin test
ATN: Acute tubular necrosis
aPTT: Activated partial-thromboplastin time
PT: Prothrombin time
PD1: Programmed cell death 1
TMA: Thrombotic microangiopathy
CAPS: Catastrophic antiphospholipid syndrome
DIC: Disseminated intravascular coagulation
IVIG: Intravenous immunoglobulin
FIB: Fibrinogen
